# On the Neuroprotective Effects of Naringenin: Pharmacological Targets, Signaling Pathways, Molecular Mechanisms, and Clinical Perspective

**DOI:** 10.3390/biom9110690

**Published:** 2019-11-03

**Authors:** Zeinab Nouri, Sajad Fakhri, Fardous F. El-Senduny, Nima Sanadgol, Ghada E. Abd-ElGhani, Mohammad Hosein Farzaei, Jen-Tsung Chen

**Affiliations:** 1 Student’s Research Committee, Faculty of Pharmacy, Kermanshah University of Medical Sciences, Kermanshah 6714415153, Iran; zeinab7641@yahoo.com; 2 Pharmaceutical Sciences Research Center, Health Institute, Kermanshah University of Medical Sciences, Kermanshah 6734667149, Iran; pharmacy.sajad@yahoo.com; 3 Biochemistry division, Chemistry Department, Faculty of Science, Mansoura University, Mansoura 35516, Egypt; biobotany@gmail.com; 4 Department of Biology, Faculty of Sciences, University of Zabol, Zabol 7383198616, Iran; Sanadgol.n@gmail.com; 5 Department of Physics and Chemistry, School of Pharmaceutical Sciences of Ribeirao Preto, University of Sao Paulo, Ribeirao Preto 14040-903, Brazil; 6 Department of Chemistry, Faculty of Science, University of Mansoura, Mansoura 35516, Egypt; dr_ghada_emad@yahoo.com; 7 Department of Life Sciences, National University of Kaohsiung, Kaohsiung 811, Taiwan

**Keywords:** neurodegenerative diseases, naringenin, pharmacological targets, signaling pathways, molecular mechanisms, drug delivery systems

## Abstract

As a group of progressive, chronic, and disabling disorders, neurodegenerative diseases (NDs) affect millions of people worldwide, and are on the rise. NDs are known as the gradual loss of neurons; however, their pathophysiological mechanisms have not been precisely revealed. Due to the complex pathophysiological mechanisms behind the neurodegeneration, investigating effective and multi-target treatments has remained a clinical challenge. Besides, appropriate neuroprotective agents are still lacking, which raises the need for new therapeutic agents. In recent years, several reports have introduced naturally-derived compounds as promising alternative treatments for NDs. Among natural entities, flavonoids are multi-target alternatives affecting different pathogenesis mechanisms in neurodegeneration. Naringenin is a natural flavonoid possessing neuroprotective activities. Increasing evidence has attained special attention on the variety of therapeutic targets along with complex signaling pathways for naringenin, which suggest its possible therapeutic applications in several NDs. Here, in this review, the neuroprotective effects of naringenin, as well as its related pharmacological targets, signaling pathways, molecular mechanisms, and clinical perspective, are described. Moreover, the need to develop novel naringenin delivery systems is also discussed to solve its widespread pharmacokinetic limitation.

## 1. Introduction

Neurodegenerative diseases (NDs) encompass several disorders, including Alzheimer’s disease (AD), Parkinson’s disease (PD), amyotrophic lateral sclerosis (ALS), Huntington’s disease (HD), multiple sclerosis (MS) and, spinal cord injury (SCI) [1]. They are considered disabling conditions that affect millions of people worldwide [2]. Although the exact etiology of these chronic diseases is entirely unknown, the critical nerve degeneration-related pathologies including decompensated neuronal structure/function changes [3], disruption in the synthesis of neurotransmitters [4], as well as abnormal protein accumulation have been clarified [5]. From the pathophysiological point of view, the ubiquitin-proteasome system and autophagy pathway, play a pivotal role in degradation and elimination of malformed proteins and damaged organelles. Disruption of these two crucial intracellular proteolytic pathways is involved in the pathogenesis of NDs [6,7]. In addition to these pathologies, mitochondrial dysfunction, oxidative insults, and neuroinflammation in the microglial cells are proposed to be implicated in neurodegeneration [8]. Microglial cells are monocyte-like immune cells which are considered as the guardian of the nervous system. There are two phenotypes of microglial cells depending on the activation state, either classical M1 or alternative M2 phenotype [9]. The activated M1 microglial cells express toll-like receptors (TLRs) as well as other pro-inflammatory cytokines such as interferon γ (INF-γ), interleukin 1β (IL-1β) and tumor necrosis factor α (TNF-α) promote the axonal degeneration and apoptotic death of neuronal cells [9]. On the other hand, M2 microglial cells express anti-inflammatory mediators such as IL-4, IL-10, IL-13, transforming growth factor β (TGF-β) and neurotrophic factors leading to neurogenesis, angiogenesis, and oligodendrogenesis (neuronal cells) and remyelination (formation of new myelin sheath around demyelinated axons) [10,11]. Therefore, the balance between M1/M2 keeps homeostasis of the immune response [9]. Naringenin could promote a phenotypic shift of microglial polarization from M1 to M2 through down-regulation of M1 markers including TNF-α and IL-1β as well as overexpression of arginase-1 (Arg-1) and IL-10 which are markers of M2 [12].

Due to the complex pathophysiological mechanisms behind the neurodegeneration, neuroprotective therapies of NDs have remained a clinical challenge. It raises the need to investigate novel multi-target agents. Natural products extracted from medicinal plants or fruits showed promising activities in treating different types of NDs through targeting multiple signaling pathways. Therefore, there is a priority in research (either in vitro or in vivo) to discover new naturally-derived compounds to target the pathways involved in NDs. Among natural entities, flavonoids are multi-target agents affecting different signaling pathways involved in neurodegeneration. Naringenin is a natural flavonoid with neuroprotective effects. Citrus fruits are rich with naringenin and its precursor, naringin [13]. In recent years, growing interest has been focused on the potential therapeutic activities of naringenin in neurological disorders. In spite of the promising effects of naringenin to manage NDs, there is a great challenge posed by poor bioavailability and slight accessibility to the brain. Here in this review, the role of naringenin in treating different neuronal disorders is described. The nanostructured formulations of naringenin which are being investigated to solve its widespread pharmacokinetic limitation are also discussed.

## 2. Chemistry of Naringenin and Its Sources

**Figure 1:**
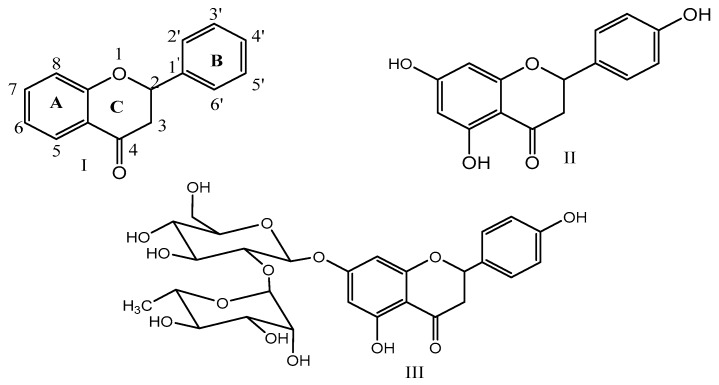
Structures of flavonoids (I); naringenin (II); and naringin (III).

Naringenin is one of the most critical naturally-occurring flavonoids (I). The basic structure of flavonoids (I) consists of three rings (A, B, and C). Naringenin (II) has a molecular formula C15H12O5 and is chemically named as 2,3-dihydro-5,7-dihydroxy-2-(4-hydroxyphenyl) 4*H*-1-benzopyran-4-one. Its molecular weight is 272.26 g·mol^−1^, and the melting point is 251 °C. This molecule is insoluble in water and soluble in organic solvents as alcohol [14]. Naringenin may be found in two forms of aglycone or its glycosidic form (naringenin-7-*O*-glucoside) [15] (Figure 1). Naringin (III) can also be seen as naringin (naringenin-7-rhamnoglucoside) [16] and narirutin (naringenin-7-*O*-rutinoside) [17]. Naringenin glycosides depending on their sugar moiety, attach via a glycosidic linkage at C7 to the flavonoid, and are cleaved by specific enzymes, then naringenin (aglycone) would be released [18].

**Figure 2:**
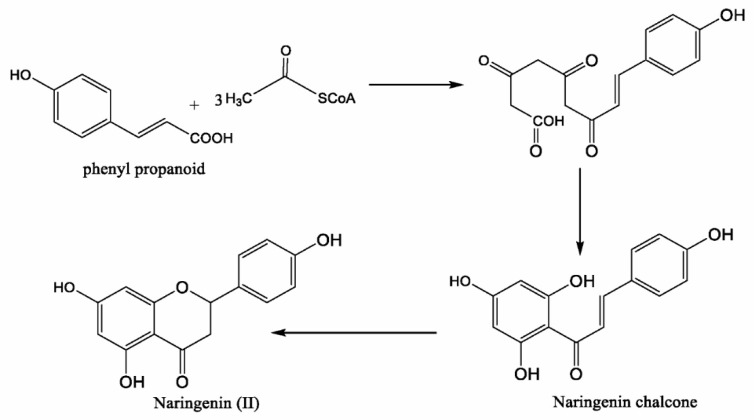
Biosynthesis of naringenin.

The biosynthesis of naringenin’s basic structure, as a flavonoid, occurs via the combination of shikimic acid and acylpolymalonate metabolic pathways. A starting compound is phenylpropane, a cinnamic acid derivative derived from shikimic acid, in which three acetate residues are incorporated followed by ring closure. The chalcone structure is intermediate to the flavone structures, which might be hydroxylated and reduced at different positions [19,20,21] (Figure 2).

Naringenin has various pharmacological properties depending on the arrangement of related functional groups in its structure. The hydroxyl groups (OH) have high reactivity against reactive oxygen species (ROS) and reactive nitrogen species. In ring (A) 5,7-*m*-dihydroxy arrangement serves to stabilize the structure after donating electrons to free radicals. The association between 5-OH and 4-oxo substituents contributes to the chelation of compounds such as heavy metals [22,23].

Naringenin has a single chiral center at carbon 2 (C2), resulting as (2*S*)- and (2*R*)-enantiomers forms which are found in citrus fruits. It has been reported to be resistant to enantiomerization over pH 9–11 [24]. Separation of the enantiomers has been explored for over 20 years [24], primarily via high-performance liquid chromatography (HPLC) on polysaccharide-derived chiral stationary phases [25,26,27,28]. There is an evidence to suggest stereospecific pharmacokinetic and pharmacodynamic profiles, which has been proposed to be an explanation for the wide variety in naringenin’s reported bioactivity [29]. Flavonols, flavones, flavanones, isoflavones, flavanols, and anthocyanidins are the most abundant flavonoids found in plants [30,31], and are potent scavengers of free radicals. As promising flavonoids, naringenin and naringin are potent antioxidants [32]; however, naringin is less potent because the sugar moiety in the former causes steric hindrance of the scavenging group. The naringenin-7-glucoside form seems less bioavailable than the aglycone form [33]. Naringenin and its glycoside has been discovered, mainly from a variety of vegetables, nuts and fruits, including grapefruit [34], bergamot [35], sour orange [36], tart cherries [37], tomatoes [38,39], cocoa [40], Greek oregano [41], water mint [42], drynaria [43], beans [44] and beverages such as coffee, tea, and red wine, as well as in medical herbs.

## 3. Naringenin in Neurodegenerative Diseases

### 3.1. Naringenin and Alzheimer’s Disease

As the most common form of NDs, AD is characterized by gradual memory decline and cognitive deficits affecting all aspects of person’s ability to do daily activities [2,45,46]. AD is accompanied by aggregation of amyloid plaques as well as neurofibrillary tangles, which are composed of amyloid-beta (Aβ) and hyperphosphorylated tau, respectively [47]. Activation of phosphokinase, glycogen synthase kinase 3β (GSK-3β), participates in the hyper-phosphorylation of tau [48]. Additionally, disrupted insulin signaling and brain insulin resistance are common in the pathogenesis of AD [49], associated with the inhibition of phosphatidylinositol-3 kinase (PI3K)/AKT pathway [50].

Currently, there is no effective treatment for AD as a neurodegenerative condition with multiple pathophysiological mechanisms. Therefore, discovering a multi-target agent is necessary to counteract AD. In this line, the anti-inflammatory, antioxidant, anti-apoptotic and neuroprotective effects [51] of naringenin make it a promising option for treating AD. Administration of naringenin interestingly improved spatial learning and memory in a rat model of AD through regulating the PI3K/AKT/GSK-3β pathway and reducing Tau hyper-phosphorylation. Additionally, naringenin improved brain insulin signaling as well as peroxisome proliferator-activated receptor gamma (PPAR-γ) [52]. Activation of PPAR-γ (belonging to the nuclear receptor superfamily) participates in insulin sensitization and increases metabolism [53].

An in vitro study in PC12 cells has shown that naringenin plays a vital role in the attenuation of apoptosis and neurotoxicity in Aβ-stimulated AD. The intracellular mechanisms responsible for anti-apoptotic activity as well as neuroprotective effects of naringenin is connected with the inhibition of caspase3, activation of PI3K/AKT, and modulation of GSK-3β signaling pathways [54].

In an Aβ-induced mouse model of AD, oral administration of naringenin resulted in the amelioration of memory deficit. Furthermore, naringenin effectively rescued the cells from apoptosis and inhibited lipid peroxidation (LPO) by decreasing hippocampal malondialdehyde (MDA) content. Besides, no significant effect was observed in behavior tasks in the co-administration of naringenin and fulvestrant as an estrogen receptor (ER) antagonist vs. naringenin alone. Therefore, neuroprotective role of naringenin may only be due to its estrogen-like activity and via the ER pathway [55].

Administration of Drynaria rhizome extract in mice alleviated memory impairment and showed considerable neuroprotective effects. Naringenin, naringenin-7-*O*-glucuronide, and naringenin-4′-*O*-glucuronide were shown to be responsible for the neuroprotective effects of Drynaria rhizome extract. However, while the three mentioned compounds could cross the BBB, just naringenin and naringenin-7-*O*-glucuronide significantly recovered Aβ-stimulated axonal atrophy in cultured cortical neurons and decreased collapsin response mediator protein-2 (CRMP2) hyperphosphorylation [56]. CRMP2 is a CNS protein that is involved in axonal and neuronal growth. The hyperphosphorylated CRMP2, which is implicated in neurofibrillary tangles, plays a pivotal role in the pathogenesis of AD-related disease. The C-terminal tail and H19 helix of CRMP-2 are more susceptible to phosphorylation by kinase enzymes such as GSK3β and cyclin depended kinase5 (CDK5) [57,58]. Therefore, these regions have been considered as an exciting drug target for AD. According to a molecular docking study, naringenin-7-*O*-glucuronide revealed the tighter binding, more affinity, and stability vs. naringenin in the active site of C-terminal tail of CRMP-2. Therefore, the presence of the glucuronyl group may play a key role in the neuroprotective effects of naringenin. Residues of Thr509, Thr514, Ser518, and Ser522 are phosphorylation sites located in the C-terminal tail of CRMP-2. Before the binding of naringenin and naringenin-7-*O*-glucuronide to the C-terminal tail, these residues have more flexibility, activity, and are more susceptible to phosphorylation. But these effects reversed after binding of naringenin and naringenin-7-*O*-glucuronide [59]. On this regard, another molecular docking study demonstrated that the occupation of CRMP2 by naringenin restricts the access of the phosphorylated enzyme to the terminal H19 helix especially in Tyr479 residue which is the susceptible residue for phosphorylation [60].

From another mechanistic point of view, acetylcholinesterase (AChE) has a crucial role in the regulation of the cholinergic system by hydrolyzing acetylcholine. An increase in AChE activity caused NDs associated with cholinergic impairment as observed in AD [61]. Methanolic extract of *Citrus junos* remarkably ameliorated AChE activity in cellular models. Naringenin is the major phytobioactive isolated from *C. junos* extract. Treating with naringenin reversed scopolamine, a muscarinic antagonist, stimulated amnesia in mice. Besides, a significant increase in spontaneous alteration behavior was observed in the mice pretreated with naringenin. The authors speculated that AChE inhibitory activity of the naringenin is responsible for promising effects of memory recovery [62].

In addition to AChE, amyloid precursor protein (APP) cleaving enzyme 1 (BACE1), and butyrylcholinesterase (BChE) are considered as potential key enzymes in the pathogenesis of AD [63,64]. As Lee et al. reported, naringenin exerted its anti-AD effects through a decrease in the activity of BACE1, AChE, and BChE in vitro [65].

In this line, Orhan et al., reported that 8-prenyl naringenin, a naringenin derivative, exhibited inhibitory effect against BChE with IC50 value of 86.58 ± 3.74 μM. According to their docking studies, 8-prenyl naringenin-BChE complex demonstrated a negative binding energy of −8.86 kcal/mol. In case of 8-prenylnaringenin, residues of BChE, including Ser198, Gly117, and His438 were responsible for forming hydrogen bonds as well as π-π stacking interaction [66].

Altogether, these findings suggest that naringenin may be considered as a multi-target and promising neuroprotective compound toward alleviating neurodegeneration observed in AD through different mechanisms.

### 3.2. Naringenin and Parkinson’s Disease

PD is a progressive neurodegenerative disorder recognized by the depletion of dopaminergic neurons in the substantia nigra pars compacta (SNpc), triggering broad loss of dopamine in the striatum. PD is characterized by activated microglia and intracytoplasmic eosinophilic proteinaceous inclusions known as Lewy bodies which are made up of alpha-synuclein self-aggregation in substantia nigra (SN) neurons [67]. Oxidative insults, dopamine depletion, and neuroinflammation play a critical role in the induction and progression of this type of NDs [68,69]. Due to the potential dopamine enhancing, potent antioxidant as well as anti-inflammatory effects of naringenin, it could be used as a beneficial agent in the treatment of PD [70]. Regarding the role of naringenin in PD, Lou and colleagues showed that administration of naringenin (70 mg/kg bwt, p.o.) in mice protected them against 6-hydroxydopamine (6-OHDA)-induced nigrostriatal dopaminergic neurodegeneration and oxidative damage through activation of the nuclear factor E2-related factor 2/antioxidant response element (Nrf2/ARE) and its downstream target genes including heme oxygenase-1 (HO-1), and glutathione cysteine ligase regulatory subunit. In addition, naringenin blocked apoptotic pathway through the inhibition of the phosphorylation of c-Jun *N* terminal kinase (JNK)/p38 and abrogating caspase 3 [71].

From another point of view, astroglia cells play a supportive role in neuronal survival by producing different types of neurotrophic growth factors [72]. Recently, Wang et al. demonstrated that naringenin (50 µM, five days) improved the release of brain-derived neurotrophic factor (BDNF) and glial cell line-derived neurotrophic factor (GDNF) as astroglial neurotrophic factors from dopaminergic neurons (in neuron-glia culture) through the up-regulation of Nrf2 [73].

Another study by Zbarskt et al. investigated the use of naringenin in the amelioration of unilateral 6-OHDA infusion in adult Sprague–Dawley male rats. Naringenin treatment protected the tyrosine hydroxylase (TH)-positive cells from damage and increased the level of dopamine and its metabolites [74].

Genetic alterations in α-synuclein, parkin, leucine-rich repeat kinase 2 (LRRK2), PTEN-induced putative kinase 1 (PINK1) and DJ-1 have also been correlated with the development of PD. The genetic modification of related genes leads to the accumulation of toxic substances and oxidative stress-inducing striatal dopaminergic terminals degeneration and formation of the aggregates containing ubiquitin and *α*-synuclein [75]. Angeline et al. investigated the ameliorative effects of naringenin (10 mg/kg/for 10 days) in rotenone-treated rats. Their study showed the ability of naringenin to improve the motor skills and body weight via the overexpression of protective proteins including parkin, PARK 7 protein (DJ1), TH and C terminus Hsp70 interacting protein (CHIP) and reduction of the level of caspase and ubiquitin [76].

Aggregation and deposition of α-synuclein, the incidence of oxidative stress, as well as neuroinflammation, has been linked to the dopaminergic neurons loss [77]. Naringenin (100 mg/kg bwt, p.o.) potentially ameliorated 1-methyl-4-phenyl-1,2,3,6-tetrahydropyridine (MPTP)-induced dopaminergic degeneration. This effect was attributed to a decrease in α-synuclein, a restoration of the reduced dopamine and its metabolites, 3,4-dihydroxyphenylacetic acid (DOPAC) and homovanillic acid (HVA), a decrease in proinflammatory cytokines (TNFα and IL1β), and an increase in superoxide dismutase (SOD) [78]. A recent mechanistic in vitro study in human neuroblastoma cells (SH-SY5Y) showed that naringenin attenuated 1-methyl-4-phenylpyridinium (MPP+)-induced dopaminergic degeneration via decreasing ROS level, abolishing α-synuclein aggregation, as well as reducing neuroinflammation by decreasing the level of TNF-α and nuclear factor-κB (NF-κB). Moreover, naringenin reduced the level of apoptosis-induced by MPP^+^ through the downregulation of Bax and overexpressed Bcl-2. Additionally, naringenin pretreatment increased the transporter for dopamine and the rate-limiting enzyme in dopamine synthesis TH [79]. Besides, naringenin interestingly abrogated MPTP-induced PD by inhibiting LPO and incrementing of catalase (CAT) and glutathione reductase (GR) and also improve locomotion efficiency. Additionally, pretreatment with naringenin in C57BL/6J mice down-expressed inducible nitric oxide synthase (iNOS) and alleviated cytoplasmic vacuolation and nuclear pigmentation in the mid-brain region [80].

Considering the attained results, naringenin could be a hopeful agent to combat PD through the modulation of several pathological pathways as well as the activation of protecting processes.

### 3.3. Naringenin and Neuroinflammation

Neuroinflammation is defined as an extreme dysregulation of the inflammatory response in the central nervous system (CNS) [81]. Neuroinflammation is mediated by the activation of microglia cells in CNS [82,83]. Overactivation of microglia mediated-infection or injury caused by innate immunity and release of various pro-inflammatory cytokines contribute to neuronal cell damage as well as the progression of NDs [84]. Naringenin targets several inflammatory signals involved in the neuroinflammation, including mitogen-activated protein kinase (MAPK), suppressor of cytokine signaling 3 (SOCS-3), NF-κB and signal transducer and activator of transcription-1 (STAT-1) [12,85,86,87]. As NFκB is known as an essential transcription factor that regulates various pro-inflammatory gene expression, naringenin induced a significant decrease in the phosphorylation and nuclear translocation of P65, a subunit of NFκB [87]. In other words, naringenin prevented the phosphorylation and degradation of IκB-α and decreased the expression of NFκB, confirming the potential anti-neuroinflammatory activity of naringenin through the suppression of NFκB [88]. From another point of view, naringenin significantly decreased neuroinflammation by reducing the phosphorylated JNK, extracellular-signal-regulated kinase (ERK1/2) [12], p38MAPK, Akt [12], and STAT-1 [87], pathways that play a principle role in the pathogenesis of neuroinflammation.

In the other study, naringenin ameliorated the levels of NO and prostaglandin E2, which indicated the inhibition of inflammation-associated enzymes, iNOS and cyclooxygenase-2 (COX-2), respectively [86], in a concentration-dependent manner, thereby decreasing neuroinflammation triggered by lipopolysaccharide (LPS) in BV-2 microglia cells. Besides, significant upregulation of the SOCS3 was observed in the cells treated with naringenin compared to small interfering RNA which was used as downregulated SOCS3 [89,90]. Administration of naringenin significantly increased the phosphorylation of AMP-activated protein kinase α (AMPKα) and protein kinase C δ (PKCδ) at Thr172 and Thr505 respectively [91,92]. Besides, in vivo results revealed that naringenin significantly recovered motor impairment and normalized morphology of activated microglia cells. Taken together, coordination between SOCS3 and AMPKα/PKCδ is of great importance in the molecular mechanism of naringenin [85].

As reported by Santa-Cecília et al., naringenin decreased IL-1β and TNF-α, as pro-inflammatory cytokines, and also reduced the expression of chemokine monocyte chemoattractant protein-1 (MCP-1) just at the gene transcription level [87].

Hence, naringenin combats against neuroinflammatory mediators and pathways, being a promising nutraceutical agent for the treatment of inflammatory related disorders.

### 3.4. Naringenin and Multiple Sclerosis

MS is an inflammatory and autoimmune disease in the CNS, which is characterized by progressive loss of neuronal myelin and disruption in neuronal function [93,94]. The first stage of MS progression is a self-reaction of T-cells against myelin antigens, which cause axonal demyelination, subsequently leading to clinical disability [85]. An imbalance between Th1 and Th2 secretes various pro-inflammatory and anti-inflammatory cytokines, respectively, accompanied by free radical production, which plays a critical role in the pathogenesis of MS [95,96].

As a multi-target agent, naringenin ameliorates the clinical manifestation of MS due to its ability to alleviate inflammatory signaling pathways, to increment antioxidant performance and to regulate the autoimmune response. Wang et al. revealed that naringenin diminished the infiltration of immune cells (T-cells, B-cells, neutrophils, macrophages), which is implicated in the CNS demyelination. Naringenin also downregulated CD4(+) T-cell differentiation Th1, Th9, Th17, as triggers of inflammatory processes, and reversed regulatory T cell (Treg) depletion, as modulator of inflammatory response, in the mice immunized with myelin oligodendrocyte glycoprotein (MOG)35–55 peptide. Naringenin blocked Th1, Th9, Th17 related transcription factors, including T-bet, PU.1, and RORγt, respectively. It also decreased the level of pro-inflammatory cytokines such as TNF-α and IL-1 [97].

Another mechanistic study by Wang et al. revealed that naringenin remarkably decreased CD4^+^ T-cell proliferation vs. CD8^+^ T-cell. Besides, Th1 cell response (IFNγ production) was more influenced by naringenin compared to Th2 cell response (IL4 production). Furthermore, naringenin could abrogate the expression of STAT3, which is the specific upstream signaling transducer RORγt and also inhibited STAT1 as well as STAT4 which are considered as corresponding signal transducers t-bet [98]. Besides, naringenin blocked IL-6-stimulated inhibition of Treg. There is also a direct link between secretion of TGF-β and generation of Treg cells as well as inhibitory effect of Treg cells on autoimmune responses [99]. TGF-β itself augmented the expression of intracellular protein Smad2/3, leading to the upregulation of T-reg transcription factor forkhead box P3 (Foxp3) [100]. Contradictory, slight mitigation of Smad2/3 expression as well as diminished Foxp3 levels were observed in cells treated with naringenin. Therefore, naringenin can regulate the development of CD4^+^ T cell lineages by impacting their respective modulatory signals [101].

Niu et al. explored the immunosuppressive effect of naringenin and its possible mechanisms on mouse T cells. Treatment with naringenin significantly blocked the proliferation of mouse T cells either induced by anti-CD3/CD28 or autoantigen MOG35-55. Besides, naringenin significantly decreased Th1 and Th17 cells-mediated cytokines (IFN-γ and IL-17A respectively) as well as pro-inflammatory cytokines, including TNF-α, and IL-6. Naringenin not only arrested T cells at G0/G1 phase in a dose depending manner but also enhanced the level of P27 which is an inhibitor of CDK [102]. Increasing the proportion of CDK4/6 is necessary for the phosphorylation and inactivation of retinoblastoma protein, which is considered as a negative regulator of cell cycle, subsequently leading to promote cell cycling from G0/G1 to S phase transition. Naringenin suppressed the phosphorylation of retinoblastoma protein, thereby modulating the immune system in MS [103]. Treatment with naringenin also decreased the level of IL-2, IL-2R α-chain (CD25) and also inhibited STAT5 phosphorylation in IL-2-restimulated activated T cells [104].

In addition, the overexpression of adhesive molecules in vascular endothelial of CNS and their interaction with related ligands on the surface of T cells, permit the T cells to attach the endothelium cells and to develop experimental autoimmune encephalomyelitis (EAE) [105]. Naringenin effectively attenuated vascular cell adhesion molecule-1 (VCAM-1) as well as its related ligand VLA4 and also downregulated CXCL10 [97,106].

From another mechanistic point of view, as the epidermal growth factor receptor ErbB4 plays a decisive role in the proliferation and survival of oligodendrocytes [107], its upregulation is involved in the remyelination and development of nerve cells [108]. In this line, the tyrosine kinase domain of ErbB4 and extracellular region of ErbB4 receptor was selected as the target receptor for molecular docking study. Joshi et al., reported naringenin as a promising candidates for activating ErbB4 receptor [109].

Therefore, the results asserted that naringenin might be a promising agent with a prosperous future in the treatment of MS, through affecting multiple signaling pathways.

### 3.5. Naringenin and Cognitive Deficit

Cognitive decline is considered as one of the most important hallmarks of various neurological diseases. Oxidative stress, inflammation, disturbance in insulin signaling, and mitochondrial dysfunction play a crucial role in the progression of cognitive deficit and dementia [52,110].

Intracerebroventricular (ICV) injection of streptozocin (STZ) causes the brain glucose depletion, and the impairment of energy metabolism, accompanied by mitigation of cholinergic neurotransmitter [111], could be related to diabetes mellitus-associated dementia [112]. Naringenin displays antioxidant, antihyperglycemic, as well as cholinergic effects, and seems to be a promising agent in the treatment of diabetes-related memory dysfunction [113,114]. According to Rahigude et al. reports, chronic administration of naringenin (50 mg/kg bwt/day, p.o.) for 58 days, significantly improved memory dysfunction, decreased hyperglycemia, mitigated oxidative stress as well as AChE activity in rats with type-2 diabetes [115]. In another study, naringenin potentially improved learning and memory dysfunction, as well as cognitive deficits caused by ICV-STZ in rats [116].

In addition, Zaki and colleagues illustrated the role of AChE activity and oxidative stress as two major causes of dementia [117]. Animals receiving naringenin revealed a decline in 4-hydroxynonenal (4-HNE), MDA, thiobarbituric acid-reactive substances (TBARS) [118], hydrogen peroxide (H_2_O_2_), protein carbonyls and an increase in glutathione (GSH) [118], glutathione peroxidase (GPx), GR, GST, SOD, CAT and Na^+^/K^+^ ATPase activity as well as upregulation of choline acetyltransferase [119,120]. Hippocampal levels of Nrf2, SOD, CAT, and GSH significantly augmented in a group treated with naringenin at a dose of 100 mg/kg. In contrast, the hippocampal level of MDA and AChE activity were diminished [110]. Naringenin also ameliorated the brain level of 5HT [120] and norepinephrine (NE); however, no effects were observed on the levels of dopamine and GABA content [118]. Therefore, the neuroprotective effect of naringenin against scopolamine-induced dementia maybe associated with its auspicious cholinergic and antioxidant activity as well as change in brain neurotransmitter content.

Neuroapoptosis and neuroinflammation play a key role in the induction and progression of memory and cognitive deficit, as isoflurane (an anesthetic) does [121]. Pre-treatment with naringenin (100 mg/kg bwt/day, p.o.) significantly attenuated neuroapoptosis and cognitive dysfunction in rats, through the downregulation of Bad, caspase-3, Bax and up-expression of Bcl-2, Bcl-xL, a decline in TUNEL and the activation of PI3K/Akt/GSK-3β pathway [122]. Besides, naringenin significantly down expressed phosphatase and tensin homolog (PTEN), as a negative modulator of PI3K/Akt/GSK-3β signaling pathway, in a dose depending manner [123].

To combat against neuroinflammation in cognitive dysfunctions, naringenin decreased the NF-κB-related inflammatory cascades by decreasing TNF-α, IL-6, IL-1β. Naringenin pretreatment also prevented cognitive deficit stimulated by isoflurane through the suppression of the NF-κB signaling pathway [123]. As reported by Khajevand-Khazaei et al., naringenin alleviated LPS-induced neuroinflammation through the downregulation of iNOS, TNF-α, COX2, NF-κB, and TLR4 [110]. TLR4 is a main upstream signaling protein that exerts a pivotal role in activation of NF-κB and subsequently upregulation of NF-κB signaling cascade including inflammatory cytokines and/or mediators (TNF-α, COX2, iNOS) [124]. From another point of view, due to the crucial role of glial fibrillary acidic protein (GFAP) in neuroinflammation, and presence of a direct link between overexpression of GFAP and hyperactivity of astrocytes [125], attenuating GFAP is of great importance. The abatement of GFAP by naringenin represents its alleviation effect on astrogliosis [110].

Recently, Sarubbo and colleagues demonstrated that naringenin (20 mg/kg bwt/day, i.p.) augmented the age-induced monoaminergic neurotransmitters depletion (noradrenaline, serotonin, and dopamine), decreased NF-κB levels, and increased tryptophan hydroxylase and SIRT1 (belonging to the histone deacetylase class III family) levels in the hippocampus. Moreover, SIRT1 contributed to a decline in inflammatory responses by suppressing NF-κB signaling pathway, thereby improving brain plasticity and memory [126,127]. It has also been reported that treatment with naringenin (50 mg/kg bwt/day, i.p.) exhibited a positive effect on cognitive function in young male rats [120].

In other studies, the ability of methyl mercury (MeHg) has been reported in the induction of severe mitochondrial dysfunction, oxidative insult, and neuronal damage, which ultimately coupled with memory and cognitive deficit [128,129,130]. Pretreatment with naringenin reversed MeHg-induced pyramidal cell damage, mitigated oxidative stress condition (confirmed by a significant increase in GSH, and GST accompanied with a marked decrease in MDA and protein carbonyl level) and ameliorated mitochondrial dysfunction (as indicated by preservation activities of mitochondrial complex I–IV and abrogation of lesions /10kb) [131].

Overall, the findings indicated that naringenin combats oxidative stress, neuroinflammation, and neuroapoptosis to overcome cognitive dysfunction thereby demonstrating the therapeutic potential.

### 3.6. Naringenin and Neurotoxicity

Excessive exposure to the neurotoxicant agents including neurotransmitter glutamate, metals (iron, sodium tungstate, aluminum) as well as, hypoxia participates in brain and spinal injury as well as widespread neurobehavioral changes [132,133,134,135]. Hypobaric hypoxia is involved in neuronal loss and behavioral dysfunction by inducing excessive formation of ROS. Hypoxia plays a causative role in triggering apoptotic pathway and upregulation of hypoxia-inducible factor 1α (HIF 1α) and its main target protein, vascular endothelial growth factor (VEGF) [123]. Enhanced free radicals and depleted antioxidants levels occur following neurotoxicity and is subsequently linked to the development of NDs [136]. Due to free radical scavenging and anti-inflammatory properties, naringenin is an attractive candidate for the management of neurotoxicity. Pre-administration of naringenin attenuated behavioral impairment in hypoxia exposed mice. Besides, naringenin alleviated hypoxia-induced oxidative stress and apoptosis by down-expression of HIF1α, VEGF as well as blocking caspase-3 and ubiquitin. Moreover, naringenin elevated the levels of parkin and chip, as ubiquitin E3 ligases or accumulated proteins in NDs [137]. In addition, naringenin improved survival of mouse neuroblastoma cells following carbaryl-induced toxicity, reduced oxidative stress (by mitigating ROS), and suppressed apoptosis (by inhibiting Bax, caspase-3 and upregulating Bcl-2). It also restored a depletion of mitochondrial membrane potential induced by carbaryl [138].

Moreover, naringenin exhibited neuroprotective effects against iron-induced neurotoxicity and anxiety-like behavioral deficit by attenuation of ROS formation, enhancement of endogenous antioxidant capacity (GSH, CAT, SOD), upregulation of AChE activity and augmentation of ectonucleotidase enzymes (such as adenosine triphosphate diphosphohydrolase and 50-nucleotide enzyme) which are responsible for regulation of extracellular ATP and adenosine concentrations in the synaptic cleft. Naringenin significantly recovered the decreased level of mitochondrial complex I–V enzymes activities and mitochondrial membrane potential, thereby reducing neurotoxicity [139].

In another similar study, co-administration of naringenin and iron potentially abolished oxidative insult markers, including MDA, NO, ROS formation, and protein carbonyls content. Naringenin also significantly enhanced enzymatic/non-enzymatic antioxidant activities, accompanied by an increase in AchE as well as Na^+^/K^+^ ATPase activity in the cerebral cortex of iron-exposed rats. In addition, co-treatment of naringenin and iron ameliorated apoptosis damage corroborated by decrease in DNA fragmentation induced by endogenous endonucleases. Moreover, naringenin ameliorated the morphological changes (necrotic cells accompanied with pyknotic nucleus and vacuolated spaces) induced by iron [140].

As reported by Sachdeva and colleagues, co-administration of naringenin and *N*-acetylcysteine (NAC) for three months significantly restored biogenic amines (dopamine, 5-HT, and NE) in a rat model of sodium tungstate-induced neurological alterations. In their study, co-administration of naringenin and NAC also reduced the oxidative stress through increasing GSH and suppressing TBARS in the brain [141].

The neuroprotective effects of naringenin against glutamate and related down-stream molecular mechanisms were examined. Naringenin potentially inhibited excitotoxicity and abolished apoptosis by reducing caspase 3 and calpain1 (Ca2^+^-dependent protease), known as caspase-independent pathway [142,143]. Phosphorylated Akt and ERK were significantly upregulated in primary culture of mouse hippocampal neurons treated with naringenin, thereby favoring neuronal survival and showing a vital role in the regulation of apoptosis [144].

From another mechanistic view, brain fatty acid-binding protein 7 (FABP7) plays a critical role in the remyelination process [145]. Naringenin provided a neuroprotective activity against oseltamivir-induced neurotoxicity via improvement of neurophysiological factors (increase in total antioxidant capacity, Ca ATPase, and FABP7) and suppression of oxidative stress through inhibiting total oxidant capacity, total nitrite oxide, as well as cytochrome P450 activation [146].

It could be concluded that naringenin can be considered as a good alternative to chemical medicines against neurotoxicants agents.

### 3.7. Naringenin and Other Neurodegenerative Diseases

Reports have revealed an auspicious role for naringenin to improve other NDs, including ischemic stroke, brain injury, HD, ALS, and SCI.

Ischemic stroke is defined as an obstruction of arteries in particular middle cerebral artery, which is responsible for supplying blood to the brain. Immediately after an ischemic stroke attack, decompensated neuronal death and damage occur [147]. Several factors including oxidative stress, apoptosis, edema-mediated by ionic imbalance as well as inflammation are implicated in the pathogenesis of ischemic stroke [148,149,150]. Naringenin, with neuroprotective properties, is an excellent therapeutic candidate for ischemic brain injury [151]. Bai et al. revealed that pre-treatment with naringenin (100 mg/kg), significantly alleviated neurological impairment, decreased the infarct size and attenuated brain water content in a rat model of permanent middle cerebral artery occlusion (pMCAO)-induced cerebral ischemia. In their study, naringenin remarkably abolished the expression of nucleotide-binding oligomerization domain (NOD2), receptor interacting protein-2 (RIP2), NF-κB, and matrix metallopeptidase 9 (MMP-9). Whereas the expression of claudin-5, protein responsible for tight junction, was elevated [152]. Belonging to the cytosolic NOD-like receptor family, NOD2 is implicated in cerebral ischemic injury by upregulation of downstream proteins, including RIP2 and NF-κB, which are considered as pivotal contributors to the initiation of the pro-inflammatory cascade [153].

A further mechanistic study by Raza et al. demonstrated that naringenin enhanced the depleted antioxidant markers, SOD and GSH. NF-κB-mediated pro-inflammatory factors including COX-2, iNOS, IL-1β as well as TNF-α were significantly alleviated in the naringenin pre-supplementation group [154]. The expression of GFAP and microglia (Iba-1) were noticeably mitigated in naringenin pre-treated group as compared to MCAO group [155]. There is a close link between enhancing expression of glial fibrillary acidic protein (GFAP) and cerebral ischemia [154].

In another study, a novel synthesized co-drug combination of naringenin and lipoic acid, named “VANL-100” was evaluated in both in vitro and in vivo models of oxidative stress. VANL-100 resulted in a significant neuroprotection against hypoxia (100-fold more potent than naringenin alone). This effect might be due to a substantial increase in intracellular antioxidant capacity by VANL-100. In an in vivo model of ischemia-reperfusion injury following transient occlusion of the middle cerebral artery (tMCAO), pre-administration of either naringenin or conjugated formulation significantly abrogated infarct size. The potency of the novel formulation was 10,000-fold compared to naringenin alone [156].

Due to the importance of oxidative stress in the occurrence of brain injury, combating oxidative mediators are very important. Nrf2, which is a prominent modulator of the oxidative stress system, plays a pivotal role in the preservation of the mitochondrial function through elevated transcription of ARE-dependent antioxidant genes [157,158]. Mitochondria dysfunction causes deadly results for neurons due to the strong dependence of neuronal survival and function on produced energy by mitochondria [159]. Discovering new antioxidant agents that regulate the Nrf2/ARE pathway may be an innovative strategy for combating oxidative stress-associated disease. Wang et al. showed that hypoxia/re-oxygenation cells treated with a high dose of naringenin (80 µM) suppressed oxidative stress by attenuating ROS generation and MDA content, as well as increasing endogenous enzymatic/non-enzymatic antioxidants such as SOD and GSH activities. Besides, naringenin treatment effectively upregulated Nrf2 downstream genes including HO-1 as well as NAD(P)H Quinone dehydrogenase1(NQO1) [160]. These findings revealed that the Nrf2/ARE pathway displayed protective effects against neuronal dysfunction.

Additionally, naringenin blocked apoptosis by increasing cell viability, accompanying with downexpression of cleaved caspase-3 protein as well as Bax protein and upregulation of Bcl-2 protein. Naringenin exerted protective effects against mitochondrial dysfunction, a primary endogenous ROS-rich source, via increasing the levels of adenylates (AMP, ADP, ATP), adenine nucleotide transporter (ANT), as a exchanger of ADP/ATP in the mitochondrial membrane, and also improvement of mitochondrial membrane potential (Δψm) [161].

A mechanistic study by Wang et al. evaluated the neuroprotective effects of naringenin in vitro on primary cultured rat cortical neurons exposed to oxygen-glucose deprivation/reoxygenation (OGD/R) and also in vivo in rats subjected to MCAO and reperfusion (MCAO/R) injury. They reported that naringenin diminished the apoptosis-related protein Bcl2, and apoptosis-related gene including Kelch-like ECH-associated protein 1, HO-1, and NQO1. It significantly reversed the translocation of Nrf2 from cytoplasm to nucleus in the primary culture of rat hippocampal neurons exposed to OGD/R. The overexpression of Nrf2 induced cell proliferation and abrogated apoptosis [162]. Naringenin and the overexpression of Nrf2 remarkably recovered OGD/R-induced mitochondrial dysfunction by increasing mitochondrial membrane potential. Besides, MCAO/R rats treated with naringenin demonstrated lower brain water content and also attenuation of the neurological impairment. Additionally, naringenin inhibited neuronal apoptosis in vivo by alleviating the number of TUNEL-positive cells [163].

As another neurodegenerative disorder with neuropathy complications, diabetes-mediated retinopathy is characterized by vascular damage accompanied by neuronal degeneration, which caused visual dimming and blindness [164]. Overproduction of free radicals has a central role in retinal cell injury [165]. Naringenin with antioxidant, antidiabetic, and anti-apoptosis effects, is a beneficial therapeutic option for combating diabetes mediated-retinal neurodegeneration [166]. Naringenin supplementation decreased the level of TBARS, as a marker of oxidative insult to lipid, protein and DNA peroxidation [167] and improved GSH level in the STZ-induced diabetic rats. Ameliorated apoptosis in the retina of diabetic rats treated with naringenin is suggested to be mediated by suppression of caspase-3, pro-apoptotic Bax as well as overregulation of anti-apoptotic Bcl-2 protein. Naringenin also restored the diminished level of BDNF, a class of neurotrophin protein, affected nerve survival and proliferation following binding with its selective receptor (tropomyosin-related kinase B (TrkB)) [168], and also amplified synaptophysin which is necessary for releasing neurotransmitter and synaptic integrity [169]. Downregulation of TrkB exacerbated apoptosis and developed neuronal injury [170]. Based on these results, it can be concluded that naringenin might be a promising candidate for prevention of diabetic retinopathy.

As other NDs, polyglutamine (polyQ) diseases are defined as an unfolding or a misfolded in the particular proteins, then leading to the aggregation of abnormal proteins in neuronal cells [171]. The endoplasmic reticulum signaling pathway has a critical role in the induction of polyQ-related disease [172]. Several studies have reported that an enhanced level of glucose-regulated protein (GRP78), a chaperon located in the endoplasmic reticulum, causes alteration in apoptosis and polyQ-related disease, such as HD [173]. HD is characterized by the accumulation of expanded polyQ tract (consists of >40 repeat of glutamine unit in huntingtin proteins) [174]. *Glycyrrhizae radix* elevated the activity of GRP78 promoter in vitro. Naringenin is the main active ingredient present in *G. radix* and disrupted the aggregation of enhanced green fluorescent protein with a pathological-length polyQ tract (EGFP-polyQ97) by overexpression of GRP78 [174,175]. Therefore, naringenin can be effective in the prevention of neurodegenerative disorders with polyQ expansion.

As a fatal neurodegenerative condition, ALS is characterized by paralysis and death within three to five years from the day of appearance of symptoms due to the impairment of respiratory systems [176,177]. The loss of bulbar and limb function is the main feature of ALS. A recent statistics study showed that around 10% of patients inherited the disease, while 90% of patients have no family history of ALS [178]. The investigation of the genetic alterations in ALS patients revealed that 20% of the familial ALS (FALS) is due to the mutations in cytosolic Cu/Zn-SOD leading to the accumulation of free radicals. The accumulation of free radicals was found to damage the homeostasis of mitochondria, the transport of axons and dysfunction in glutamate transporter [179]. Considering the critical role of naringenin in combating oxidative stress and glutamate-induce neurotoxicity, naringenin could be a promising agent in ALS patients to improve their survival rate.

As another neurodegenerative condition, SCI is a devastating and debilitating condition that resulted in neurologic deterioration accompanied by sensory-motor deficit [143]. Inflammation, apoptosis, and oxidative stress contribute to the secondary damage of SCI [180]. Pharmacological interventions that mitigate the secondary phase process may provide the therapeutic efficacy for SCI [181]. Shi et al. indicated that the administration of naringenin ameliorated SCI by down-regulating mir-223, and decreasing tissue myeloperoxidase, as a marker of neutrophil infiltration, as well as abrogating inflammatory cytokines [182].

**Figure 3:**
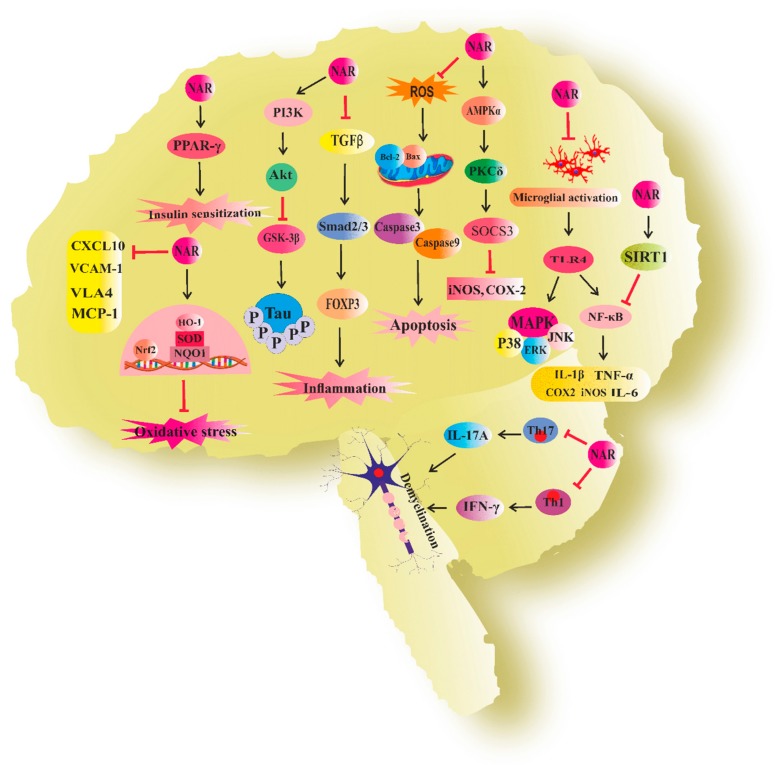
Neuroprotective mechanisms of naringenin. NAR, naringenin; PPAR-γ, peroxisome proliferator-activated receptor gamma; CXCL10, C-X-C motif chemokine ligand 10; VLA4, very late antigen 4; VCAM-1, vascular cell adhesion molecule-1; MCP-1, monocyte chemoattractant protein-1; Nrf2, nuclear factor E2-related factor 2; HO-1, hemoxygenase-1; SOD, superoxide dismutase; NQO-1, NAD(P)H quinone dehydrogenase1; PI3K/AKT, phosphoinositide 3-kinase/AKT; GSK3-β, glycogen synthase kinase3-β; TGF-β, transforming growth factor-β; Foxp3, forkhead box P3; Treg, regulatory T cell; Th1, T helper1; iNOS, inducible nitric oxide synthase; COX-2, cyclooxygenase-2; SOCS-3, suppressor of cytokine signaling 3; AMPKα, (AMP)-activated protein kinase α; PKCδ, protein kinase Cδ; JNK, c-Jun *N* terminal kinase; ERK, extracellular-signal-regulated kinase; MAPK, mitogen-activated protein kinase; TNF-α, tumor necrosis factor α; INF-γ, interferon γ; IL-1β, interleukin 1β; NF-κB, nuclear factor-κB; TLR4, toll-like receptor 4; ROS, reactive oxygen species.

Altogether, naringenin has shown a bright future in the treatment of NDs. Figure 3 demonstrates the molecular mechanisms underlying the neuroprotective effect of naringenin for combating NDs (Figure 3).

## 4. Nanostructured Formulations of Naringenin for Management of Neurodegenerative Diseases.

Naringenin is a well-known antioxidant and anti-inflammatory flavanone with wide ranges of beneficial effects in the management of NDs but there are several challenges for the accessibility and accumulation of naringenin in the brain. Poor ability of naringenin to cross biological membranes, its poor water solubility, and high first-pass effect, as well as related gastrointestinal degradation, cause low bioavailability and limit its clinical applications [183].

One of the promising ways to overcome these challenges is to involve nanostructured formulations of naringenin. Several nanoformulations have been developed for naringenin to increase its bioavailability, solubility, and decrease drug degradation. The most investigated nanoformulations are based on biodegradable and biocompatible polymers that encapsulate naringenin in nanostructures including solid lipid nanoparticles, nanocapsules, nanomicelles, nanoliposomes, and nanoemulsions [14]. Md et al. investigated the potential neuroprotective effect of naringenin-based nanoemulsion against Aβ-induced AD. SHSY5Y cells pretreated with either naringenin or nanoemulsions showed attenuated phosphorylation of tau, decreased β amyloid-stimulated ROS production and abolished BACE [184]. BACE is an enzyme that breaks the APP and ultimately leads to aggregation of Aβ and induction of amyloidogenic pathway [185]. While pretreatment with naringenin was not able to hinder the production of Aβ, pretreatment with naringenin-nanoemulsion alleviated the level of tau [184], highlighting the importance of using nanoformulations in drug delivery systems.

To achieve an efficient management of PD in animal models, intranasal delivery of vitamin E-loaded naringenin nanoemulsion was successfully used. Intranasal administration of nanoemulsion of naringenin accompanied by levodopa played an essential role in the amelioration of behavioral parameters, increment of GSH and SOD, as well as decrement of MDA level in 6-OHDA-induced PD in rats. Synergism between the antioxidant effects of naringenin and vitamin E, lipophilicity, nanoscale size of nanoemulsion as well as intranasal administration of nanoemulsion which provided high concentration of naringenin in brain through olfactory pathway caused better efficacy of nanoemulsion compared to naringenin alone [186].

In another study, treatment of human mesenchymal stem cells with naringenin encapsulated into gelatin-coated polycaprolactone nanoparticle conferred neuroprotective effects on OGD-stimulated ischemic stroke [187]. The nanoformulation showed a sustained release manner and contributed to blood-brain barrier integrity as well as cellular morphology improvement. The nanoparticles significantly abrogated NF-κB activation as a trigger of inflammatory cascade. Besides, remarkable decrease in pro-inflammatory factors such as TNF-α, IFN-γ, and IL-1β along with a reduction in inducible enzymes, including COX2 and iNOS was observed in cells treated with the nanoparticle vs. OGD group [188]. Thus, the nano-formulation may be a suitable candidate to be evaluated in an animal model of ischemic stroke.

Overall, several findings suggested that nanostructured formulations of naringenin were able to overcome limitations affecting the beneficial effect of naringenin in NDs. All of the current data focused on the safety and efficacy of naringenin loaded nanoparticles in vitro and/or in vivo models of NDs. Future preclinical and clinical trials for mentioned nanostructured formulations of naringenin should be carried out to corroborate the therapeutic effect and promote health in the patients with NDs.

## 5. Conclusions

In spite of exhaustive research, no effective treatment has been investigated for NDs. The complex pathophysiological mechanisms of neurodegeneration, along with the lack of safe and efficient therapies for NDs, raise the need to find novel multi-target treatments. Nowadays, with a priority in research, natural products, as well as their active compounds, have attained special attention in the treatment of NDs. Among natural products, naringenin is a multi-target flavonoid, possessing promising neuroprotective effects, through targeting multiple therapeutic targets and signaling pathways (Table 1). Although, numerous in vitro and in vivo studies have elucidated the ability of naringenin to alleviate several types of NDs, the lack of clinical trials regarding therapeutic potential of naringenin is a critical limitation. There exists a dire need to conduct well-designed clinical trials in patients with NDs. On the other hand, the poor bioavailability and slight brain accessibility of naringenin, has resulted in a lack of efficacy in clinical trials. To solve these drawbacks, nanostructured formulations of naringenin have also been developed, and naringenin based nanoparticles exhibited biocompatibility and biodegradability characteristics as well as high bioactivity. Further studies and engineered methods are necessary to provide surface modification of nanoformulations of naringenin to optimize drug delivery to CNS.

A further area of research on the safety and efficacy of naringenin and its nanoformolations in human, as well as related novel pathological signaling pathways of NDs in the human, will show new avenues in the prevention, management, and treatment of several diseases.

## Abbreviation

iNOSinducible nitric oxide synthaseCOX-2cyclooxygenaseSOCS-3suppressor of cytokine signaling 3AMPKα(AMP)-activated protein kinase αPKCprotein kinase CJNKc-Jun *N* terminal kinaseERKextracellular-signal-regulated kinaseMAPKmitogen-activated protein kinaseTNF- αtumor necrosis factor αINF-γinterferon γIL-1βinterleukin 1βMCP-1monocyte chemoattractant protein-1STAT-1signal transducer and activator of transcription-1PI3K/Aktphosphatidylinositol-3 kinase/AktERestrogen receptorROSreactive oxygen speciesMDAmalondialdehydeBACEβ-Site amyloid precursor protein (APP) cleaving enzymeAPPamyloid precursor proteinGSK-3βglycogen synthase kinase 3βPPAR-γperoxisome proliferator-activated receptor gammaAchEacetylcholinesteraseRIP2receptor interacting protein-2NF-κBnuclear factor-κBMMP-9matrix metallopeptidase 9SODsuperoxide dismutaseGSHglutathioneNrf-2/AREnuclear factor E2-related factor 2/ antioxidant response elementHO-1hemoxygenase-1NQO-1NAD(P)H quinone dehydrogenase1TBARSthiobarbituric acid-reactive substancesBDNFbrain-derived neurotrophic factorTrkBtropomyosin-related kinase BGRP78glucose-regulated proteinFoxp3forkhead box P3DOPACdihydroxyphenylacetic acidHVAhomovanillic acidTHtyrosine hydroxylaseCHIPC terminus Hsp70 interacting proteinLPOlipid peroxidationGDNFglial cell line-derived neurotrophic factorHIF1ahypoxia inducible factor1aVEGFvascular endothelial growth factor5-HT5-hydroxytryptamineGFAPglial fibrillary acidic protein6-OHDA6-hydroxydopamineICV-STZintracerebroventricular-streptozotocineADAlzheimer’s diseasePDParkinson’s diseaseLPSlipopolysaccharideMeHgmethyl mercuryMPTP1-methyl-4-phenyl-1,2,3,6-tetrahydropyridineMPP^+^1-methyl-4-phenylpyridiniumpMCAOpermanent middle cerebral artery occlusiontMCAOtransient middle cerebral artery occlusionEAEexperimental autoimmune encephalomyelitis

## Figures and Tables

**Table 1:** Neuropharmacological mechanisms of naringenin against different type of neurodegenerative diseases.

Type of Diseases	Method	Model	Neuropharmacological Mechanisms and Outcome	References
	LPS Induced neuroinflammation	In vitro: BV2 microgelia cells	↓iNOS ↓COX-2 ↑SOCS3 ↑ AMPKα ↑ PKCδ	[85]
Neuroinflammation	Induced by LPS	In vitro: BV2 microgelia cells	↓JNK ↓ERK ↓p38 ↓MAPK ↓TNF-α ↓IL-1β ↑ Arg-1↑IL-10	[12]
	Induced by LPS	In vitro: BV2 microgelia cells	↓TNF-α ↓IL-6 ↓IL-1β ↓MCP-1 ↓NfκB ↓MAPK ↓Akt ↓iNOS ↓ COX-2	[86]
	Induced by LPS/ IFN-γ	In vitro: microglia	↓p38 MAPK ↓ERK1/2 ↓STAT1 ↓iNOS ↓ TNF-α	[87]
	Aβ25-35-induced AD	In vitro: PC12 cells	↓apoptosis, ↓caspase3, ↑ PI3K/AKT, ↑ER	[54]
AD	Aβ25-35-induced AD	In vivo: Wistar rats	↓MDA ↓apoptosis, ↑ER, ↑spatial memory and cognition	[55]
	ICV-STZ- induced AD Induced by Aβ1-42 and Aβ25-35	In vivo: Sprague–Dawley rats In vitro: cultured cortical neurons In vivo: 5XFAD Mice	↓Tau hyper-phosphorylation, ↑ PI3K/AKT ↓GSK3-β ↑ PPAR- γ ↑insulin signaling ↑spatial learning and memory ↓amyloid plaques, ↓Tau hyper-phosphorylation	[52] [56]
Amnesia	Induced by scopolamine	In vivo: ICR mice	↓AchE activity ↑spontaneous alteration behavior	[62]
	pMCAO- induced cerebral ischemic	In vivo: Sprague–Dawley rats	↓infarct size, ↓brain water content, ↓ NOD2, RIP2, NF-κB, MMP-9, ↑ claudin-5	[152]
	Induced by hypoxia	In vitro: neurons isolated from the brain of Sprague–Dawley rats	↓ROS, MDA, ↑SOD, GSH↓caspase-3, Bax, ↑ Bcl-2, ↑AMP, ADP, ATP, ANT↑ Nrf2, HO-1, NQO1	[160]
Ischemic stroke	MCAO/R-induced ischemic stroke	In vivo: Sprague–Dawley rats	↓brain water content, ↓TUNEL-positive cells	[163]
	Induced by MCAO/R	In vivo: Wistar rats	↓infarct size, neurological deficits, brain water, ↑ motor, and somatosensory function ↑SOD, GSH, MPO, TBARS, ↓COX-2, iNOS, ↓IL-1β, TNF-α ↓ NF-κB	[155]
Diabetic retinopathy	STZ-induced diabetic retinopathy	In vivo: Wistar albino rats	↓ TBARS, ↑GSH, ↓caspase-3, Bax, ↑Bcl-2 ↑ BDNF, TrkB, synaptophysin,	[169]
Polyglutamine diseases	-	In vitro: mouse C3H10T1/2 cells, COS-7 cells, and HeLa-tetQ97 Cells	↑GRP78	[175]
	(MOG)35-55-induced EAE	In vivo: C57BL/6 mice	↓ Th1, Th9, Th17, ↑ Treg, ↓T-bet, PU.1, and RORγt,	[97]
EAE	Induced by anti-CD3/CD28	In vivo: C57BL/6 mice	↓IFNγ, ↓STAT1, STAT3, STAT4, ↓IL-6, ↑gp-130, ↓Foxp3	[101]
	Induced by anti-CD3/CD28 and (MOG)35-55	In vitro: mouse T cells	↓T cells proliferation, ↓ IFN-γ, IL-17A ↓ TNF-α, IL-6, block T cells at G0/G1 phase ↑ P27, ↓ retinoblastoma protein phosphorylation, ↓IL-2, CD25 ↓ STAT5	[104]
	Induced by 6-OHDA	In vitro: Human neuroblastoma SH-SY5Y cells. In vivo: C57BL/6 mice	↑Nrf2/ARE ↑HO-1, ↓ROS ↑GSH ↓ JNK and p38	[71]
PD	MPTP-induced PD	In vivo: C57BL/6J mice	↓α-synuclein ↑dopamine transporter ↑DOPAC ↑HVA ↑TH ↓TNFα & IL1β ↑SOD	[78]
	Rotenone-induced PD	In vivo: Wistar rats	↓ubiquitin and caspase3 improvement of motor skills ↑parkin ↑CHIP ↑PARK 7 protein ↑TH	[76]
	MPTP-induced PD	In vivo: C57BL/6J mice	↑GRx & CAT ↓LPO& iNOS ↓ nuclear pigmentation and cytoplasmic vacuolation	[80]
	-	In vitro: primary rat midbrain neuron-glia co-cultures	↑ BDNF, GDNF↑ Nrf2 ↑Dopaminergic neurons survival	[73]
	6-OHDA-induced PD	In vivo: Sprague-Dawley rats	↑DOPAC, ↑HVA, ↑Dopamine↑TH	[74]
	Induced by MPP^+^	In vitro: Human neuroblastoma SH-SY5Y cells	↓ ROS ↓NF-κB ↓TNF-α ↓Bax ↑Bcl-2	[79]
	Induced by sodium tungstate	In vivo: Wistar rat	↑GSH ↓ROS ↓ TBARS ↑Dopamine	[141]
	Induced by glutamate	In vitro: primary culture of mouse hippocampal neurons	↑ Erk1/2 & Akt phosphorylation ↓calpain-1 & caspase-3	[144]
Neurotoxicity	Induced by hypobaric hypoxia	In vivo: Swiss albino mice	↓HIF1a ↓VEGF ↓caspase-3 ↓ ubiquitin ↑CHIP ↑ parkin	[137]
	iron-induced neurotoxicity	In vivo: Wistar rat	↑ SOD, CAT ↓ROS ↑ AChE ↓MDA ↑Na^+^/K^+^ ATPase	[139]
	Induced by oseltamivir	In vivo: Wistar rat	↑FABP7 ↑Ca ATPase, ↑TAC ↓TOC ↓TNO ↓ cytochrome P450	[146]
	Induced by iron	In vivo: Wistar rat	↓ROS ↑GSH, CAT, SOD ↑AchE ↑ectonucleotidase enzymes ↑mitochondrial complex I–V enzymes ↑ mitochondrial membrane potential	[140]
	Induced by carbaryl	In vitro: mouse neuroblastoma cells	↓ROS ↓Bax, caspase-3 ↑Bcl-2 ↑mitochondrial membrane potential	[138]
	Induced by ICV-STZ	In vivo: Wistar rats	↑ Learning and memory performance	[116]
	Induced by ICV-STZ	In vivo: Wistar rats	↑learning & memory ↓TBARS, MDA, 4-HNE, H2O2, protein carbonyl, ↑GSH, SOD, CAT ↑Na^+^/K^+^ ATPase activity	[119]
	Induced by scopolamine	In vivo: albino Wistar rats	↓AChE ↑GSH ↓TBARS ↓TNFα ↓5HT, NE ↑spontaneous alternation performance & conditioned avoidance response	[118]
	Induced by isoflurane	In vivo: Sprague–Dawley rats	↓Bad, caspase-3, Bax ↑ Bcl-2, Bcl-xL ↓TUNEL ↑ PI3K/Akt ↓PTEN ↓NF-κB, TNF-α, IL -6, IL-1β, Improvement of cognitive dysfunction	[123]
	Induced by LPS	In vivo: albino Wistar rats	↓TLR4, NF-κB, TNF-α, COX2 and iNOS ↑Nrf2, SOD, CAT, and GSH ↓MDA and AChE ↓GFAP ↑ spatial recognition memory, discrimination ratio & retention and recall capability	[110]
Cognitive deficit	Age-induced cognitive deficit	In vivo: Sprague–Dawley rats	↑ SIRT1 ↓ NF-κB ↑serotonin, noradrenaline, dopamine, TH	[126]
	Induced by MeHg	In vivo: Swiss Albino mice	↑ mitochondrial complex I- IV activities, ↓lesions /10kb ↑GSH, GST ↓MDA & protein carbonyl ↑spatial and recognition memory	[131]
	-	In vivo: young adult male Albino Wistar rats	↓AChE, ↑ 5HT	[120]
	Induced by type 2 diabetes mellitus	In vivo: Young Sprague–Dawley rats	↓AChE, ↓hyperglycemia ↑memory performance	[115]
